# Somatic comorbidity, metabolic syndrome, cardiovascular risk, and CRP in patients with recurrent depressive disorders

**DOI:** 10.3325/cmj.2013.54.453

**Published:** 2013-10

**Authors:** Radmila Topić, Davor Miličić, Zoran Štimac, Mladen Lončar, Vedran Velagić, Darko Marčinko, Miro Jakovljević

**Affiliations:** 1 Department of Psychiatry, University of Zagreb School of Medicine, University Clinical Centre Zagreb, Zagreb, Croatia; 2 Department of Cardiology, University of Zagreb School of Medicine, University Clinical Centre Zagreb, Zagreb, Croatia

## Abstract

**Aim:**

To investigate the association between depression, metabolic syndrome (MBS), somatic, particularly cardiovascular comorbidity, and low-grade chronic inflammation assessed using C-reactive protein (CRP).

**Methods:**

This cross-sectional study included 76 patients with recurrent depressive disorder (RDD) and 72 non-depressed medical staff controls from the Department of Psychiatry, University Hospital Center Zagreb between January 2011 and June 2012.

**Results:**

Seventy-five percent of patients had somatic comorbidity. The most common comorbid conditions were cardiovascular disorders (46.1%), locomotor system diseases (35.5%), carcinoma (15.8%), thyroid diseases (9.2%), and diabetes (9.2%). MTB was more common in RDD patients (31.6%) than in controls (23.6%), but the difference was not significant. Elevated CRP was found to be significantly more frequent in patients with recurrent depressive disorders (RDD) (35.5%; χ^2^ test, *P* = 0.001, Cramer V = 0.29) than in controls (12.5%) and was associated with lowered high-density lipoprotein and overweight/obesity.

**Conclusion:**

We found some intriguing links between stress, depression, metabolic syndrome, and low grade inflammation, which may be relevant for the prevalence of somatic comorbidity in patients with RDD, but further studies are needed to confirm our results.

Depression and somatic, particularly cardiovascular disorders, are closely interrelated, which is why it is important to study depression, metabolic syndrome, and somatic comorbidity together. Depressed mood is recognized to contribute to the development and progression of some somatic, especially cardiovascular diseases, while cardiovascular and some other somatic diseases may increase the risk of depression. In spite of a considerable progress in comorbidity research, this phenomenon is one of the greatest epistemological, research, and clinical challenges to contemporary psychiatry and medicine (1,2). In clinical practice, comorbidity is underrecognized, underdiagnosed, underestimated, and undertreated. The definition of comorbidity and multisystem disorder is also not very clear. Do depression, diabetes, and coronary heart disease represent comorbid disorders or a multisystem mind-body disease (2)? In addition to high rates of somatic comorbidity, depression is characterized with high rates of metabolic, oxidative stress, and pro-inflammatory risk-factors. The relationship of depression with future coronary heart disease (CHD) has been a important research topic over the last 20 years and has provoked much thought on the link between the mind, brain, and heart (3,4).

Metabolic syndrome (MBS) characterized by metabolic abnormalities, such as abdominal obesity, high blood pressure, hyperglycemia, low high density lipoprotein (HDL) cholesterol, and elevated triglycerides is an important risk factor for CHD and type 2 diabetes mellitus (DM). Depression, which is also an independent risk factor for CHD and DM type 2, is frequent in individuals with MBS (5), and MBS is more common in depressed patients than in general population (6). However, it is still not clear whether and to what extent MBS is a significant mediator in the depression-CHD comorbidity. Therefore, the MBS-depression relationship is of significant scientific and public health interest.

Growing evidence indicates that low grade chronic inflammation may be associated with MBS and coronary heart disease (5), as well as with depression (7), but the exact nature of this relationship is still unknown (8). Depression is often characterized with high levels of C-reactive protein (CRP), which may contribute to cardiovascular disease development in depressed patients without somatic diseases (7). On the other hand, elevated CRP levels are significantly correlated with oxidative stress markers and an increased risk of MBS, and individuals with MBS may have a higher inflammation status and a higher level of oxidative stress.

The aim of this study was to investigate the prevalence of somatic, particularly cardiovascular comorbidity, and MBS and its components in patients with recurrent major depression, as well as the prevalence of increased CRP as an additional comorbidity risk factor. This report has been part of a bigger multidimensional study, which includes many other parameters like cortisol, cathecholamines, homocisteine, methylenetetrahydrofolate reductase polymorphism, HbA1C, ECG, as well as A/B and D personality types.

## Participants and methods

### Participants

The sample comprised 148 participants, 76 patients with recurrent depressive disorder (without psychotic features) and 72 controls (medical staff). The study was conducted at the Department of Psychiatry, University Hospital Center Zagreb, between January 2011 and June 2012. Diagnosis of recurrent depressive disorder was established by the ICD-10 and DSM IV criteria by the attending psychiatrist. The mean age of the participants was 50.98 ± 8.4 years, 51.42 ± 8.5 years in patients with depression and 50.51 ± 8.3 years in controls. The groups did not differ in age and sex. Written informed consent was obtained from all participants. The study protocol was approved by the Ethics Committee of the University Hospital Center, Zagreb.

### Measures

*Sociodemographic and anamnestic questionnaire.* Sociodemographic and anamnestic questionnaire was designed for the purpose of this study to assess the following variables: age, sex, education, employment, marital status, number of children in family, onset of illness, personal somatic anamnesis, psychiatric heredity and comorbidity, consumption of alcohol, and cigarette smoking.

*Biological parameters – metabolic syndrome and CRP.* MS was defined according to NCEP ATP III criteria (National Institute of Health, Third Report of the National Cholesterol Education Program Expert Panel on Detection, Evaluation, and Treatment of High Blood Cholesterol in Adults), which require that three or more of the following five criteria are present: 1) increased waist circumference: >88 in women or 102 in men; 2) elevated triglycerides: ≥1.7 mmol/L; 3) reduced high-density lipoprotein (HDL) cholesterol: <1.03 mmol/L in men or <1.29mmol/L in women; 4) elevated blood pressure: ≥130/85 mm/Hg; 5) elevated fasting glucose: ≥5.6 mmol/L.

Waist circumference was measured by tape measure and blood pressure was determined by pressure gauge. Venipuncture was performed every morning after 12 hours of overnight fasting. After collection, blood samples were delivered to the central laboratory and serum concentration of triglycerides, total cholesterol, HDL cholesterol, low density lipoprotein cholesterol (LDL-c), and serum glucose were determined using enzyme methods and commercial kits (Olympus Diagnostic, GmbH, Hamburg, Germany) on Olympus AU 600 automated analyzer. CRP serum levels were determined using immunoturbidimetric method on Olympus AU 2700. The cut-off point for elevated CRP was set at 5 mg/L.

### Statistical analysis

Normality of distribution of Framingham score results was tested by Kolmogorov-Smirnov test. Results are presented as medians and interquartile ranges. Differences between two groups in continuous variables were tested using Mann-Whitney test, with area under the curve (AUC) given as the standardized measure of size effect. AUC was calculated according to the formula: U/(m × n), where U is the result of the Mann-Whitney test, m and n are sizes of two samples. Differences between nominal variables were analyzed by χ^2^ test, with Cramer V as the standardized measure of size effect. Fisher exact test was used for analysis of two dichotomous variables, with Cramer V as the standardized measure of size effect. To test simultaneous occurrence of key metabolic risk factors, Jaccard similarity measure was used. The level of significance was set to *P* < 0.05, with 95% confidence intervals. All the analyses were carried out using SPSS 17.0 (SPSS Inc., Chicago, IL, USA) statistical software package.

## Results

Significantly more patients with RDD than controls had a physical disease (75.0% vs 19.4%; χ^2^ test, *P* < 0.001, Cramer V = 0.56). There were also significantly more RDD patients than controls with diseases of locomotor system (35.5% vs 6.9%; χ^2^ test, *P* < 0.001, Cramer V = 0.35), as well as carcinoma (25.8% vs 1.4%; χ^2^ test, *P* = 0.002, Cramer V = 0.25), diabetes (9.2% vs 0%; χ^2^ test, *P* = 0.014, Cramer V = 0.22), and dyslipidemia (7.9% vs 0%; χ^2^ test, *P* = 0.028, Cramer V = 0.2). Significantly more RDD patients (46.1%) than controls had some CV disease (13.9%) (χ^2^ test, *P* < 0.001, Cramer V = 0.35) (Table 1).

**Table 1:** Physical and cardiovascular diseases by groups

	Experimental group		Control group		*P*; effect
n	%	n	%	
Physical diseases*					
diseases of the locomotor system	27	35.5	5	6.9	<0.001; 0.347
carcinoma	12	15.8	1	1.4	0.002; 0.254
thyroid disease	7	9.2	4	5.6	0.535
diabetes	7	9.2			0.014; 0.217
gastritis/ulcer	4	5.3	2	2.8	0.682
dyslipidemia	6	7.9			0.028; 0200
osteoporosis	2	2.6			0.497
dermatitis	2	2.6			0.497
anemia	1	1.3			>0.999
asthma	1	1.3			>0.999
other	20	26.3	3	4.2	<0.001; 0.306
no diseases	19	25.0	58	80.6	
Cardiovascular diseases*					
yes	35	46.1	10	13.9	<0.001; 0.349
no	41	53.9	62	86.1	
total	76	100	72	100	
Number of diseases^†^					
none	19	25.0	58	80.6	<0.001; 0.19
one	32	42.1	13	18.1	
two	20	26.3	1	1.4	
three	3	3.9			
four	2	2.6			
total	76	100	72	100	
Any physical disease*					
yes	57	75.0	14	19.4	<0.001; 0.556
no	19	25.0	58	80.6	
total	76	100	72	100	

*Fisher exact test; level of statistical significance or probability of Type I error; effect = Cramer V given as the effect size for χ^2^ test, only for significant results.

†Mann-Whitney U test for number of diseases, effect = area under the curve (AUC) as the effect size for Mann-Whitney test.

Significantly more RDD patients with elevated CRP (37%) than RDD patients with normal CRP had lowered HDL (14.3%) (χ^2^ test; *P* = 0.042; Cramer V = 0.26). There was no significant difference in these parameters among controls (Table 2).

**Table 2:** Differences in metabolic disorders and metabolic syndrome prevalence by C reactive protein (CRP)

	Recurrent depressive disorders (RDD) group					Control group				
	elevated CRP		normal CRP		*P*; effect	elevated CRP		normal CRP		*P*; effect
	n	%	n	%		n	%	n	%	
Hypertension (>80, 135 mm Hg)*										
no	14	51.9	35	71.4	0.132	6	66.7	42	66.7	>0.999
yes	13	48.1	14	28.6		3	33.3	21	33.3	
total	27	100	49	100		9	100	63	100	
Hyperglycemia *										
no	20	74.1	40	81.6	0.558	5	55.6	53	84.1	0.065
yes (>6.1)	7	25.9	9	18.4		4	44.4	10	15.9	
total	27	100	49	100		9	100	63	100	
Cholesterol*										
normal	10	37.0	11	22.4	0.191	0	0.0	12	19.0	0.340
elevated (>5.0)	17	63.0	38	77.6		9	100	51	81.0	
total	27	100	49	100		9	100	63	100	
Triglycerides*										
normal	13	48.1	24	49.0	>0.999	4	44.4	40	63.5	0.467
elevated (>1.7)	14	51.9	25	51.0		5	55.6	23	36.5	
total	27	100	49	100		9	100	63	100	
High-density lipoprotein*										
normal	17	63.0	42	85.7	0.042; 0.261	5	55.6	52	82.5	0.083
lowered (<1.04)	10	37.0	7	14.3		4	44.4	11	17.5	
total	27	100	49	100		9	100	63	100	
Low-density lipoprotein*										
normal	11	40.7	15	30.6	0.451	0	0.0	10	15.9	0.343
elevated (>3.0)	16	59.3	34	69.4		9	100	53	84.1	
total	27	100	49	100		9	100	63	100	
Body mass index*										
normal	6	22.2	19	38.8	0.003; 0.402	3	33.3	26	41.9	0.033; 0.313
overweight (>25)	7	25.9	23	46.9		1	11.1	25	40.3	
obesity (>30)	14	51.9	7	14.3		5	55.6	11	17.7	
total	27	100	49	100		9	100	62	100	
Waist circumference*										
normal	8	29.6	25	52.1	0.089	5	55.6	39	61.9	0.782
increased	19	70.4	23	47.9		4	44.4	24	38.1	
total	27	100	48	100		9	100	63	100	
Metabolic syndrome*										
no	14	51.9	38	77.6	0.038; 0.265	5	55.6	50	79.4	0.201
yes	13	48.1	11	22.4		4	44.4	13	20.6	
total	27	100	49	100		9	100	63	100	
Framingham score (median, interquartile range)^†^	7.5	4-13	7	3- 11	0.524	11	4- 19	7	3- 9	0.176

*χ^2^ test; effect = Cramer V given as the effect size for χ^2^ test, only for significant results.

†Mann-Whitney U test for number of diseases, effect = area under the curve (AUC) as the effect size for Mann-Whitney test.

Obesity was significantly more common among participants with elevated CRP in both RDD (χ^2^ test, *P* < 0.003; Cramer's V = 0.40) and control groups (χ^2^ test; *P* < 0.033; Cramer's V = 0.31) (Table 2).

**Figure 1:**
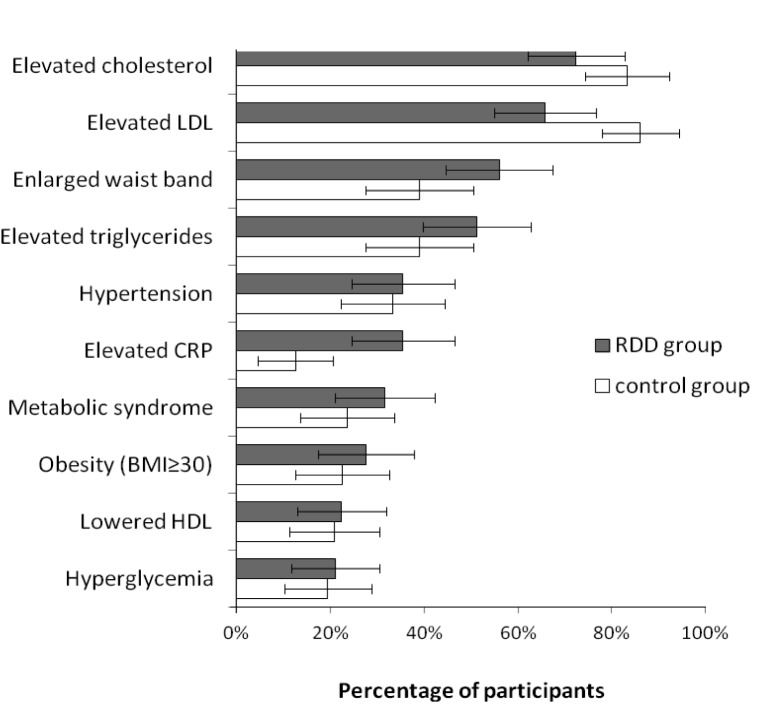
Prevalence of metabolic risk factors by groups (n_RDD_ = 76; n_control_ = 72); error bar represents 95% confidence interval; sorted by prevalence in recurrent depressive disorders (RDD) group. LDL – low-density lipoprotein; CRP – C-reactive protein; BMI – body mass index; HDL – high-density lipoprotein.

Significantly more RDD patients with elevated CRP (48.1%) than RDD patients with normal level of CRP (22.4%) had MBS (χ^2^ test; *P* = 0.038; Cramer's V = 0.27). MBS was also more frequent in controls with elevated CRP level, but the difference was not significant (Table 3) (Figure 1).

**Table 3:** Jaccard proximity measures for binarized key metabolic risk factors; only recurrent depressive disorders (RDD) patients (n = 76)

	Hypertension	Total cholesterol	LDL	Triglycerides	HDL	HbA1c	BMI	Metabolic syndrome	CRP
Hypertension	1.00	0.19	0.19	0.18	0.00	0.16	0.21	0.28	0.18
Total cholesterol	0.19	1.00	0.81	0.54	0.13	0.30	0,.9	0.34	0.26
LDL	0.19	0.81	1.00	0.41	0.12	0.30	0.29	0.35	0.26
Triglycerides	0.18	0.54	0.41	1.00	0.22	0.21	0.36	0.47	0.27
HDL	0.00	0.13	0.12	0.22	1.00	0.17	0.23	0.28	0.29
HbA1c	0.16	0.30	0.30	0.21	0.17	1.00	0.36	0.30	0.28
BMI	0.21	0.29	0.29	0.36	0.23	0.36	1.00	0.67	0.41
Metabolic syndrome	0.28	0.34	0.35	0.47	0.28	0.30	0.67	1.00	0.34
CRP	0.18	0.26	0.26	0.27	0.29	0.28	0.41	0.34	1.00

*Abbreviations: LDL – low-density lipoprotein; CRP – C-reactive protein; BMI – body mass index; HDL – high-density lipoprotein.

To check for simultaneous occurrence of key metabolic risk factors, Jaccard similarity measure was used. The highest rate of simultaneous occurrence was found among blood lipids: Jaccard similarity measure for elevated total cholesterol and elevated LDL was 0.81 and for tryglicerides and total cholesterol 0.54. CRP and BMI also showed a somewhat higher similarity measure (0.41).

## Discussion

To our knowledge, this is the first study that investigated the prevalence of comorbid somatic, particularly cardiovascular disorders, metabolic syndrome, and elevated CRP in patients with recurrent major depression. Our results demonstrated that patients with RDD were at increased risk of physical, particularly cardiovascular comorbidity and that the prevalence of somatic comorbidity was 75%. In other words, physical diseases were found to be almost four times more frequent in depressed patients than in controls. This finding is similar to that of 67.1% in patients with bipolar disorder (9). The most common comorbid conditions in our RDD patients were cardiovascular disorders (46.1%), locomotor system diseases (35.5%), carcinoma (15.8%), thyroid diseases (9.2%), and diabetes (9.2%). MTB was present in 31.6% of RDD patients. This is in line with a study that found the prevalence of MTB in depressed population to be 8%-41% (10). The data on the relationship between depression and metabolic syndrome are very controversial due to inconclusive results, variations in study design, definitions of MTB, evaluation of depressive symptoms or depressive disorder, and overlapping of recurrent depression and bipolar II disorder (10-12). A systematic review and meta-analysis of epidemiological studies indicated a bidirectional association between depression and metabolic syndrome (12). Depression is considered as a risk factor for MTB or even one of the components of MTB (13), while on the other hand MTB may be a risk factor for depression. Recently, it has been reported that MTB may be a mediator in the pathway between depression and CVD (14). Elevated hs-CRP, a sensitive and dynamic marker for low grade systemic inflammation, has been recognized as a risk factor for development and prognosis of cardiovascular diseases, cardiovascular events in type 2 diabetes, and adverse cardiac events in depressed patients with CHD (7). Our results confirmed that patients with RDD had evidence of low grade systemic inflammation, which may be implicated in the development of comorbidities, particularly cardiovascular. Elevated CRP was found to be significantly more frequent in patients with RDD than in controls. Other studies also indicate an association between depression and increased activity of acute phase proteins and inflammatory molecules, including CRP (15,16). In our study, the prevalence of MTB was more than two times higher in participants with elevated CRP than in those with normal CRP, but the difference was significant only in depressed patients. It seems that inflammatory processes and metabolic syndrome may represent a link between depression and different kinds of somatic comorbidity. Inflammation may be associated with MTB and its components, and MTB is sometimes even considered a proinflammatory state (17). Measurement of subclinical inflammatory markers like CRP might improve the prediction of cardiovascular disease and diabetes in depressed patients with MTB. Our result showed that elevated CRP level was associated with lowered HDL and overweight/obesity. Other studies have shown that CRP is associated with components of MTB, like abdominal obesity (17,18). Adipose tissue secretes a number of proinflammatory cytokines including interleukin 6 (IL-6), which regulates hepatic production of CRP (19). There is evidence suggesting that elevated CRP may promote atherogenesis due to its influence on the uptake of oxidized LDL cholesterol by macrophages (7).

Several limitations of our study should be noted. The main limitation is the cross-sectional design, which cannot detect the temporal changes of MTB, its components, and inflammation. Furthermore, our sample is relatively small and possibly not representative of the diverse population of patients with RDD. The control group comprised only medical personnel and future studies should include an additional control group of people with low professional stress or a group from the general population. In spite of its limitations, our study contributes to better understanding of the association between MTB, its components, low grade inflammation, stress, and depression. We found some intriguing links between stress, depression, metabolic syndrome, and low grade inflammation, which may be relevant to the prevalence of somatic, particularly cardiovascular, comorbidity in patients with RDD.
